# Biofunctional Polymer Coated Au Nanoparticles Prepared via RAFT-Assisted Encapsulating Emulsion Polymerization and Click Chemistry

**DOI:** 10.3390/polym12071442

**Published:** 2020-06-27

**Authors:** Sónia O. Pereira, Tito Trindade, Ana Barros-Timmons

**Affiliations:** Department of Chemistry, CICECO-Aveiro Institute of Materials, University of Aveiro, 3810-193 Aveiro, Portugal; sonia.pereira@ua.pt (S.O.P.); tito@ua.pt (T.T.)

**Keywords:** RAFT-assisted encapsulating emulsion polymerization, gold nanostructures, click chemistry, biosensing, Langmuir monolayers

## Abstract

The use of reversible addition-fragmentation chain transfer (RAFT)-assisted encapsulating emulsion polymerization (REEP) has been explored to prepare diverse types of colloidal stable core–shell nanostructures. A major field of application of such nanoparticles is in emergent nanomedicines, which require effective biofunctionalization strategies, in which their response to bioanalytes needs to be firstly assessed. Herein, functional core–shell nanostructures were prepared via REEP and click chemistry. Thus, following the REEP strategy, colloidal gold nanoparticles (Au NPs, *d* = 15 nm) were coated with a poly(ethylene glycol) methyl ether acrylate (PEGA) macroRAFT agent containing an azide (*N3*) group to afford *N3*–macroRAFT@Au NPs. Then, chain extension was carried out from the NPs surface via REEP, at 44 °C under monomer-starved conditions, to yield *N3*–copolymer@Au NPs–core–shell type structures. Biotin was anchored to *N3*–copolymer@Au NPs via click chemistry using an alkynated biotin to yield biofunctionalized Au nanostructures. The response of the ensuing biotin–copolymer@Au NPs to avidin was followed by visible spectroscopy, and the copolymer–biotin–avidin interaction was further studied using the Langmuir–Blodgett technique. This research demonstrates that REEP is a promising strategy to prepare robust functional core–shell plasmonic nanostructures for bioapplications. Although the presence of azide moieties requires the use of low polymerization temperature, the overall strategy allows the preparation of tailor-made plasmonic nanostructures for applications of biosensors based on responsive polymer shells, such as pH, temperature, and photoluminescence quenching. Moreover, the interaction of biotin with avidin proved to be time dependent.

## 1. Introduction

Reversible addition-fragmentation chain transfer (RAFT) polymerization is a well-known reversible-deactivation radical polymerization (RDRP) mechanism that has attracted great attention due to its versatility in controlling the composition and molecular weight (*M*_w_) of the resulting polymers using mild reaction conditions [1,2]. In the past years, this polymerization mechanism has been explored to encapsulate inorganic cores in order to obtain well-defined core–shell nanostructures via RAFT-assisted encapsulating emulsion polymerization (REEP) [3,4]. This strategy involves two main steps: first, the adsorption of a previously prepared amphiphilic polymer containing a chain transfer agent (CTA)–the macroRAFT (MR) agent, onto the inorganic core; and then chain extension via RAFT emulsion polymerization under monomer-starved conditions. The MR agent acts as a surfactant, thus allowing emulsion polymerization to be carried out without additional surfactants.

The use of REEP to encapsulate inorganic particles was first reported in 2008 in order to encapsulate pigment particles with an average size of circa 300 nm [5]. Since then, chemical and morphological distinct inorganic cores have been encapsulated, such as carbon nanomaterials, CdS, PbS, CeO_2_, SiO_2_, montmorillonite, and SiO_2_@Gd_2_O_3_:Eu^3+^ [6,7,8,9,10,11,12,13,14]. The use of REEP to encapsulate colloidal gold nanoparticles (Au NPs) with an average diameter of 15 nm has also been explored due to its potential applications in biosensing [15,16]. Au NPs exhibit localized surface plasmon resonances (LSPR) that are sensitive to the size, shape, and the surrounding environment (such as interparticle interaction and surface modification). Yet, surface modification and/or encapsulation is required to ensure colloidal stability and specific functionalities [17].

Surface-initiated polymerization is a good strategy to prepare well-controlled core–shell structures [18], and REEP has proven to be a robust strategy to provide multifunctionality to Au NPs. By the judicious selection of the monomers, the polymer shells can be tailored in order to provide stability, biocompatibility, and specific functionalities to the colloidal Au NPs. This specific functionality can be provided by functional groups (e.g., amines, carboxylic acid groups, azides, alkynes) to which molecules of interest are covalently bonded. The latter include organic dyes or biorecognition elements that are specifically recognized by target proteins for biosensing. An alternative approach to prepare well-controlled core–shell structures is the *grafting-to* approach involves the preparation of duly functionalized polymers which are subsequently grafted onto the surface of the NPs. In this case, whilst the control over the polymer composition and molar mass may be higher, the grafting density can be rather limited due to steric reasons and the slow diffusion of the polymer chains. Nevertheless, Farinha et al. have reported the successful use of this approach to prepare hybrid fibers of cellulose acetate [19].

The azide alkyne 1,3-cycloaddition catalyzed by Cu(I)–CuAAC reactions, often referred as “click chemistry”, has proven to be a powerful synthetic route to prepare functional materials. In fact, the combination of RAFT polymerization with click chemistry has been carried out via two pathways: (1) by using “clickable” RAFT agents or (2) by incorporating “clickable” monomers in the polymerization. This second strategy allows the preparation of polymers with higher functional group densities. Polymers can be also post-modified with azide groups for subsequent “click” reactions [20].

Following our previous works on the encapsulation of Au NPs via REEP at 70 °C [15,16], we decided to investigate the combination of REEP and click chemistry in order to prepare biofunctional polymer@Au NPs with core–shell structures. As such, azide-polymer@Au NPs have been prepared using REEP at 44 °C and subsequently functionalized with biotin via click chemistry. Then, the biosensing ability of the ensuing nanostructures was assessed by optical spectroscopy using avidin as bioanalyte, which is a protein that presents high affinity to biotin. Complementary studies to better understand the interactions between the copolymer, biotin, and avidin were also performed using Langmuir monolayers of the copolymer at the air/water interface, which proved that these interactions are time dependent.

## 2. Experimental

### 2.1. Reagents

Poly(ethylene glycol) methyl ether acrylate (PEGA, *M*_n_ = 480 g/mol, Sigma-Aldrich), acrylic acid (AA, 99%, Fluka), RAFT agent 2-(dodecylthiocarbonothioylthio)-2-methylpropionic acid (TTC-A, 98%, HPLC, Sigma-Aldrich), initiator 2,2′-Azobis [2-(2-imidazolin-2-yl)propane]dihydrochloride (VA-044, Wako) (VA-044, *t*_1/2_ = 10 h at 44 °C), initiator 4,4′-azobis(4-cyanovaleric acid) (ACPA, 98% Fluka), hydrogen tetrachloroaurate (III) trihydrate (HAuCl_4_·3H_2_O, 99.9+%, Sigma-Aldrich), sodium citrate tribasic dehydrate (Na_3_C_6_H_5_O_7_·2H_2_O, Sigma-Aldrich) and 1,3,5-trioxane (99.5+%, Acros) were used as received. Monomers *n*-butyl acrylate (BA, 99%, Acros) and methyl methacrylate (MMA, 99%, Acros) were passed through a column of neutral aluminum oxide (Carlo Erba, particle size 63–200 µm) prior to use to remove the inhibitor. 3-Azido-1-propanol (97%, Sigma-Aldrich), *N*-(3-dimethylaminopropyl)-*N*′-ethyl-carbodiimide (EDC, Sigma-Aldrich), *N*,*N*-dimethylaminopyridine (DMAP, Aldrich, 99%), biotin (≥99%, TLC lyophilized powder from Sigma-Aldrich), avidin (from chicken egg white, Pierce™, from ThermoFisher Scientific), bovine serum albumin (BSA, Sigma-Aldrich) and copper (II) sulfate pentahydrate (CuSO_4_·5H_2_O, JMGS) were used as received. Sodium ascorbate was prepared by the neutralization of a solution of *L*-ascorbic acid (Panreac) with sodium bicarbonate, precipitated with isopropanol, and dried at 40 °C under vacuum [21]. Prior to use, 3-butyn-1-ol (Aldrich, 93%) was bubbled for 1 h with dry nitrogen and then stored in a flask containing molecular sieves 3 Å, under dry nitrogen. *N*,*N*-Dimethylformamide (DMF, VWR AnalaR, 99.5%) was dried with molecular sieves 3 Å for 24 h before use. Ethanol and diethyl ether BDH Prolabo were purchased from VWR. Dimethyl sulfoxide-*d*_6_ ((CD_3_)_2_SO, Merck) was used to dissolve the macroRAFT agent for ^1^H-NMR subsequent characterization. Ultrapure water obtained from a MilliQ water purification system (Millipore, Billerica, MA, USA) was used throughout the work.

### 2.2. Synthesis

#### 2.2.1. Synthesis of MacroRAFT Agents

TTC-A was dissolved in ethanol; then, the monomer AA or PEGA was added followed by the initiator ACPA. 1,3,5-Trioxane was also added as internal standard for the determination of monomer conversion by ^1^H NMR using a ratio [*trioxane*]/[*monomer*] = 6. The mixture was purged with nitrogen for 30 min in an ice bath under stirring. The reaction was carried out at 70 °C during 4 h for the synthesis of P(PEGA_40_)-TTC, while for the synthesis of P(AA_2_-*co*-PEGA_40_)-TTC, first, AA was polymerized during 3 h, and then PEGA was added and the polymerization proceeded over 4 extra hours. See Table S1, in Supporting Information, for specific experimental conditions. Aliquots were withdrawn and analyzed by ^1^H NMR for the determination of monomer conversion. The final product was precipitated using cold diethyl ether. The resulting yellow viscous liquid was further purified by two additional dissolution/precipitations steps using room temperature and cold diethyl ether, respectively, and then dried under reduced pressure at room temperature.

#### 2.2.2. Preparation of N3-MacroRAFT Agent

In a 100 mL round bottom flask, the previously prepared P(AA_2_-*co*-PEGA_40_)-TTC (1.5 g) was dissolved in dichloromethane (15 mL); then, EDC (0.020 g) and DMAP (0.003 g) were added under stirring using an ice bath. The 3-azido-1-propanol (0.0153 mL) was added to the mixture, and the reaction was carried out for 2 h, upon which it was allowed to thaw to room temperature and stirred for 2 days. The azide-functionalized macroRAFT agent (*N3-macroRAFT* agent) was purified by dialysis (Spectra/Por^®^ Dialysis Membrane MWCO 6–8 kDa from SpectrumLabs.com). Dichloromethane was used as solvent during one day and replaced at least twice.

#### 2.2.3. Preparation of Au NPs by the Citrate Method (Au-Cit NPs)

Following the procedure of Turkevich et al. [22], 10 mL of sodium citrate solution (38.8 mM) were added to 100 mL of HAuCl_4_·3H_2_O solution (1 mM) previously brought to 90 °C and under vigorous stirring. After 1 h, heating was switched off, and stirring was kept overnight. Prior to use, the colloid was centrifuged for 30 min at 15,600× *g*. The supernatant was removed and replaced by an equal volume of ultrapure water. Au NPs with a diameter of circa 15 nm were obtained, and the concentration (mol NPs/L) was calculated by UV-Vis spectroscopy as described in the literature [23].

#### 2.2.4. Copolymerization of MacroRAFT Agent via RAFT Emulsion Polymerization

The diblock copolymers were prepared by RAFT emulsion polymerization. The macroRAFT agent was dissolved in water, and the pH was adjusted to 7.5 using an aqueous solution of NaOH (0.5 M). Then, the solution of initiator VA-044 (1.4 mL, 3.2 mM) was added to the reaction vessel. The reaction vessel was placed in an ice bath, and the mixture was purged with nitrogen for 30 min under stirring. The polymerization was started by placing the reaction vessel in an oil bath at 44 °C. An aliquot (100 µL) of the mixture of hydrophobic monomers (10 MMA: 1 BA *w*/*w*) was added each hour during the first 6 h of reaction, which continued for 18 more hours. After this, the reaction vessel was placed in an ice bath in contact with oxygen to stop the polymerization. Samples were periodically withdrawn to monitor the monomer conversion by gravimetric analyses and the evolution of the average particle diameter by dynamic light scattering (DLS) measurements.

#### 2.2.5. Preparation of N3-Copolymer@Au NPs via REEP

*N3*-copolymer@Au NPs were prepared using a mixture of the macroRAFT P(PEGA_40_)-TTC with a macroRAFT agent modified with the azide group (*N3*-macroRAFT agent) using a ratio of [2 macroRAFT: 1 *N3*-macroRAFT] in two steps: (1) adsorption of the macroRAFT mixture onto Au NPs to afford *N3*-macroRAFT@Au NPs, which was followed by (2) chain extension via REEP.

Regarding step (1), a solution of *N3*-macroRAFT agent (0.05 mM) and a solution of macroRAFT agent (0.05 mM) were prepared, and the pH was increased to 7–8 with the use of NaOH (0.1 M and 0.01 M). *N3*-macroRAFT agent solution (6.7 mL) was first added dropwise to a dispersion of Au NPs (2 mL, 6.0 × 10^−9^ mol NPs/L) and stirred for 2 h. Then, 13.3 mL of macroRAFT agent solution was also added dropwise, and the mixture was stirred overnight, at room temperature. The mixture was centrifuged for 30 min at 15,600× *g*, the supernatant was collected, and the precipitate redispersed in 10 mL of ultrapure water.

In step (2), the pH of the freshly prepared *N3*-macroRAFT@Au NPs (9 mL) was adjusted to pH = 8 using NaOH (0.1 or 0.01 M), and then a solution of initiator VA-044 (1 mL, 0.25 mM) and 5 µL of the mixture of hydrophobic monomers MMA:BA (10:1 *w*/*w*) were added. The mixture was purged with nitrogen for 30 min in an ice bath under stirring, and the polymerization started by placing the reaction vessel in an oil bath at 44 °C. Then, 20 µL more of MMA:BA (10:1 *w*/*w*) was added in the first 6 h (5 µL, each hour, using a microsyringe), and the polymerization continued up to a total of 24 h. The polymerization was quenched by placing the reaction vessel in an ice bath and by opening it to have contact with oxygen from the air. The resulting colloid was centrifuged 30 min at 15,600× g, the supernatant was collected, and the precipitate was redispersed in the same volume using ultrapure water.

#### 2.2.6. Preparation of Copolymer@Au NPs via REEP

Copolymer@Au NPs, without azide functional groups, were prepared following the same procedure as described above for the preparation of N3-copolymer@Au NPs, but in this case, during the adsorption step, only P(PEGA_40_)-TTC was used. A solution of macroRAFT agent (20 mL, 0.05 mM, pH 7–8) was added dropwise under stirring, to a dispersion of Au NPs (2 mL, 6.0 × 10^−9^ mol NPs/L) and stirred overnight at room temperature.

#### 2.2.7. Synthesis of Alkynated Biotin

Biotin was functionalized with an alkyne group following the work of Matyjaszewski et al. [24]. The DMF used in this synthesis was previously dried using molecular sieves and purged with nitrogen. First, 0.7 g of biotin, 1.1 g of EDC, and 0.035 g of DMAP were added to a 100 mL round-bottom flask. The contents were vacuum dried, and 49 mL of DMF was added. The flask was placed in an ice bath. Then, 0.4 g of 3-butyn-1-ol was dissolved in 4.7 mL of DMF, in N_2_ atmosphere. This solution was added slowly to the reaction vessel using a syringe pump over a period of 2 h. The mixture was allowed to reach room temperature and was stirred under nitrogen for 2 days. After this period of time, the DMF was removed using a rotary evaporator, and the contents were dissolved into 80 mL of dichloromethane. This solution was washed once with 50 mL of an aqueous solution of NaOH (1 M) and four times with 50 mL of distilled water. Dichloromethane was removed using a rotary evaporator. The resulting white-brownish solid was dissolved in THF and then precipitated twice into an excess of hexane, and the precipitate was filtered under reduced pressure. The alkynated biotin was dissolved in DMSO-*d*_6_ and characterized by ^1^H-NMR (see Figures S6 and S7 in Supporting Information).

#### 2.2.8. Preparation of Biotin-Copolymer@Au NPs via Click Reaction

First, 250 µL of alkynated biotin (1.67 mM, aqueous DMSO solution 10% to ensure full solubilization of biotin) was added to 10 mL of *N3*-copolymer@Au nanostructures, under stirring. Then, 42 µL of CuSO_4_·5H_2_O (2.5 mM) and 42 µL of sodium ascorbate (5 mM) were added, and the mixture was stirred for 24 h. The determination of azide moieties in the nanostructure was not possible; therefore, it was considered to be 8 × 10^−7^ moles (i.e., the number of moles of *N3*-macroRAFT agent used in the adsorption). Based on this, the ratios used were: 1 [azide]: 0.5 [alkyne]: 0.125 [CuSO_4_]: 0.25 [sodium ascorbate]. After 24 h, the colloidal mixture was centrifuged for 30 min at 15,600× *g*, the supernatant was collected, and the precipitate was redispersed in 10 mL using ultrapure water.

The same procedure was also applied to the copolymer@Au nanostructures (without azide moieties), which was used as control of the click reaction (“click control”). Furthermore, these nanostructures were extensively rinsed to assess the efficiency of the click reaction. Thus, three precipitation/redispersion cycles by centrifugation were performed in the colloid, using ultrapure water or an aqueous solution containing DMSO (1 DMSO:10 H_2_O *v*/*v*) in order to increase the solubility of biotin in the supernatant. The relative portions used were based of the information available in data sheets of biotin. However, an interesting study regarding the solubility of biotin in different solvents and temperatures has been reported by Chen et al. [25].

#### 2.2.9. Biosensing Tests

A solution of avidin and BSA (0.5 mg/mL) was prepared with phosphate saline buffer (PBS, pH 7.4, 10 mM). First, 10 µL of BSA or avidin solution were added to 350 µL of biotin-copolymer@Au NPs, neat copolymer@Au NPs, and copolymer@Au NPs (without azide moiety) submitted to the click chemistry reaction (named “click control”); the last two were used as controls. After 10 min, the visible spectra were recorded. As a control of the dilution, PBS was also added to the Au nanostructures.

#### 2.2.10. Preparation of Langmuir Monolayers to Study Copolymer–Biotin–Avidin Interactions

First, 600 µL of a biotin solution (0.4 mg/mL) was added to 34 µL of latex and stirred overnight. The mixture was dried under an air flow and then dissolved with an ethanol:chloroform (1:10) solvent mixture. Then, 10 µL of this solution (2 mg/mL of copolymer containing biotin) was spread on the air/water interface using ultrapure water or an aqueous solution of avidin as the subphase. The avidin solution used as the subphase was prepared by diluting 500 µL of an avidin solution (0.5 mg/mL) in 500 mL of ultrapure water. Before barriers compression started, the copolymer–biotin mixture was allowed to reach equilibrium (solvent evaporation) for 15 min. These studies were performed in collaboration with Oliveira’s group in Brazil (University of São Paulo in São Carlos) in a class 10,000 clean room and a KSV-Nima Small KN2001. Then, 10 µL of copolymer solution containing biotin, as described above, were spread at the air/water interface using an aqueous solution of avidin (0.5 mg/mL) as the subphase. Before barriers compression started, the copolymer–biotin mixture was allowed to reach equilibrium (solvent evaporation) for 15 min plus a time contact of 20 min, 1 h, 2 h, or 3 h. Langmuir monolayers were prepared by compressing the barriers at 10 mm^2^/min.

## 3. Characterization

The UV/VIS spectra of the colloids were recorded using quartz cells, a Jasco V-560 UV/VIS spectrometer, and using water as the reference. The zeta potential and dynamic light scattering (DLS) measurements were carried out using a ZetaSizer Nano ZS Model Zen 3500 from Malvern. The Au colloids were analyzed as prepared, and the latexes were 10-fold diluted. Centrifugation was performed in a Force 1618 Microcentrifuge at room temperature. Fourier-transform infrared (FTIR) spectra were acquired using a Bruker Optics Tensor 27 spectrometer coupled to a horizontal attenuated total reflectance (ATR) cell, using 1024 scans at a resolution 4 cm^−1^. ^1^H-NMR spectra were recorded on a Bruker Avance 300 spectrometer. Scanning Electron Microscopy (SEM) in transmission mode (STEM) images were obtained using a Hitachi SU-70 operating at 15 or 30 kV. The samples were prepared by placing a drop of diluted colloidal solutions on a copper grid coated with an amorphous carbon film and left to evaporate.

Gel permeation chromatography – size exclusion chromatography (GPC-SEC) analysis were performed in Department of Chemistry Engineering-DEQUI in the Engineering School of Lorena–EEL-USP, Brazil, in a GPC instrument from Waters adapted with a TDA 302 Triple Detector Array from Viscotek. To characterize the samples, THF was used as eluent with triethylamine (0.3% *v*/*v*), and a flow rate of 1.0 mL·min^−1^ was used. The samples were solubilized overnight, filtered using a polytetrafluoroethylene (PTFE) membrane (pore size of 0.45 μm), and afterwards, 100 μL was immediately injected. The separation occurred in three columns from Phenomenex with size exclusion limits of 10^3^ Å, 10^4^ Å, and 10^6^ Å. The temperature of the columns and detector was maintained at 35 °C during the analysis. The average molar masses (number-average molar mass, *M*_n_, and weight-average molar mass, *M*_w_) and the molar mass dispersity (Ð = *M*_w_/*M*_n_) were derived from the refractive index (RI) signal using a calibration curve based on polystyrene standard from Sigma-Aldrich and Viscotek.

## 4. Results and Discussion

### 4.1. Synthesis of MacroRAFT Agents

**Scheme 1:**
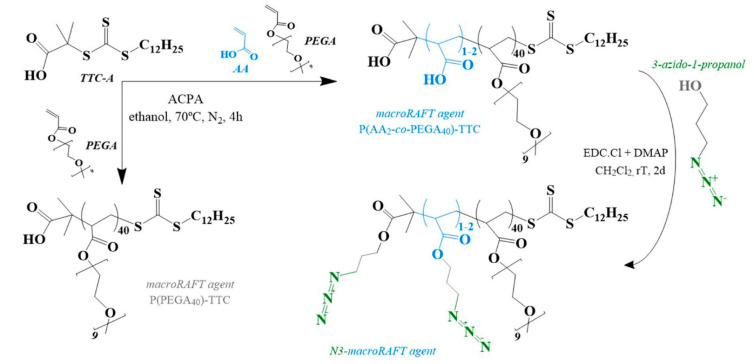
Synthesis of the macroRAFT agents. RAFT: reversible addition-fragmentation chain transfer, TTC: 2-(dodecylthiocarbonothioylthio)-2-methylpropionic.

Two types of macroRAFT agents have been synthesized in this work following a similar procedure but in which either P(PEGA_40_)-TTC or P(AA_2_-*co*-PEGA_40_)-TTC was used. The latter was subsequently modified in order to anchor an azide moiety, yielding an *N3*-macroRAFT agent (see Scheme 1). The monomer PEGA was selected because polymers such as poly(ethylene glycol) are biocompatible. Furthermore, these polymer coatings limit nonspecific interactions with proteins, [26,27,28], which could be very important for the application of surface-coated Au nanostructures as biosensors.

In order to functionalize the P(AA_2_-*co*-PEGA_40_)-TTC with the azide moiety, the macroRAFT agent was dispersed in dichloromethane, and EDC was added to activate the carboxylic groups of the AA units in the presence of 4-(dimethylamino)pyridine (DMAP). The 3-azido-1-propanol was added to the reaction vessel and stirred at room temperature over two days. 

**Figure 1:**
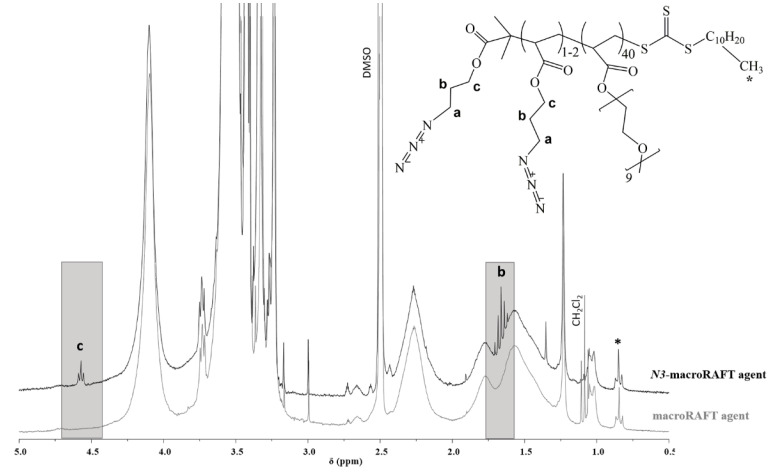
^1^H-NMR spectra of P(AA_2_-*co*-PEGA_40_)-TTC before and after functionalization with the azide moiety. (*inset*) Chemical structure of the *N3*-macroRAFT agent. PEGA: poly(ethylene glycol) methyl ether acrylate.

The *N3*-macroRAFT agent was characterized by ATR-FTIR and ^1^H-NMR spectroscopies. The new ester bond was difficult to identify by ATR-FTIR spectroscopy because its vibration frequency (1730 cm^−1^) overlaps with the ester bonds of the PEGA repeating unit of the macroRAFT agent. Nevertheless, the vibrational mode characteristic of the azide was observed at 2097 cm^−1^, as illustrated in Figure S1 in the Supporting Information. The presence of the azide was further confirmed by ^1^H-NMR spectroscopy (Figure 1), which revealed the presence of peaks at 4.57 and at 1.66 ppm corresponding to the protons from two methylene groups of 3-azido-1-propanol [29]. However, the signal of the protons associated with the CH_2_-N moiety is masked by the signals of the PEGA chains. The ratio of the integrated peaks at the chemical shifts 4.57 ppm and 0.84 ppm, corresponding to the methyl of the *Z*-group, indicates that there is one or two azide moieties per polymer chain (see details in the Supporting Information).

### 4.2. Preparation of Copolymer@Au NPs

Copolymer@Au NPs were prepared via RAFT-assisted encapsulating emulsion polymerization (REEP). This strategy requires two steps: (1) the adsorption of the macroRAFT agent onto the NPs and (2) chain extension via the emulsion polymerization of hydrophobic monomers. Due to the presence of azide moieties in this study, a radical initiator with a high decomposition rate coefficient (VA-044) was used in order to perform the emulsion polymerization step below 50 °C [30]. Although Farinha et al. [19] reported the use of higher temperature in the presence of azide moieties for the sake of safety, to prevent secondary side reactions, the use of lower temperature was considered a better option. This was further supported by the fact that azides can undergo cycloaddition reactions with the double bonds of methyl methacrylate monomers [31]. As in our previous studies, the encapsulation of Au NPs via REEP was carried out at 70 °C using ACPA as the initiator [15,16], and the effect of temperature on VA-044 on the polymerization of the hydrophobic monomers via RAFT emulsion polymerization was first studied in the absence of Au NPs.

For comparative purposes, chain extension of the macroRAFT P(PEGA_40_)-TTC was first carried out via RAFT emulsion polymerization at 70 °C using VA-044 as the initiator. The polymerization conditions, [macroRAFT]/[initiator] and [monomer]/[macroRAFT] ratios, were kept the same as in our previous work where ACPA was used as the initiator [16] as well as the mixture of hydrophobic monomers methyl methacrylate (MMA) and butyl acrylate (BA) (10:1 *w*/*w*) [15]. As expected, monomer conversion was much higher when VA-044 was used. In fact, 81% of monomer conversion was obtained after 1 h of polymerization and after 4 h, 100% of monomer conversion was reached, which corresponds to a DP_MMA:BA_ = 168. (See Figure S2A in Supporting Information). Then, a similar polymerization was carried out using VA-044 but at 44 °C. In this case, only 34% conversion was reached after 24 h; see Figure S2B in Supporting Information. When comparing the results obtained using VA-044 at 70 °C with those obtained using ACPA also at 70 °C in our previous studies [15,16], it is striking to observe that 81% monomer conversion was only obtained after 4 h of reaction, when the latter was used as an initiator. Differences in the rate of polymerization using ACPA and VA-044 at 70 °C were previously reported by Perrier et al. [32,33] and are due to the fact that VA-044 has a higher decomposition rate coefficient (*k_d_*) which allows increasing the rate of the polymerization reaction without affecting its livingness.

**Figure 2:**
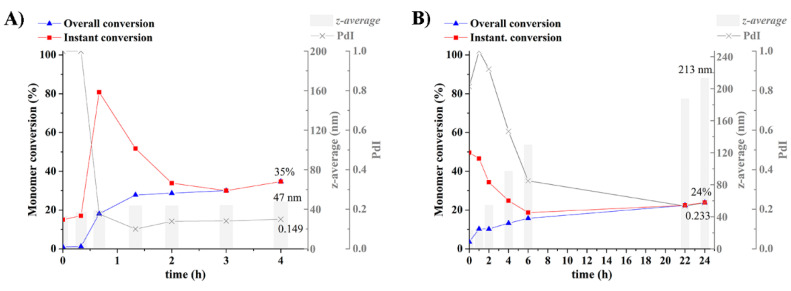
Monomer conversion (gravimetric analysis) and hydrodynamic diameter (*z*-average, dynamic light scattering (DLS) measurements) of aliquots withdrawn during the copolymerization of MMA:BA (10:1 *w/w*) in the presence of P(PEGA_40_)-TTC using the initiator VA-044 at (**A**) 70 °C and at (**B**) 44 °C. BA: butyl acrylate, MMA: methyl methacrylate.

The use of REEP to encapsulate the Au NPs requires the controlled addition of the hydrophobic monomers to guarantee the stability of the colloidal system. Additionally, it also prevents chain extension from the free macroRAFT agent present in solution, which is a competitive process in relation to the polymerization from the macroRAFT@Au NPs [15]. Therefore, besides the experiments carried out under batch conditions discussed above, the polymerizations were also studied under monomer starved conditions. In this case, at 70 °C monomer conversion was 35% and it was 24% at 44 °C. The reactions were also followed by DLS in order to monitor the evolution of particle size distribution over time (Figure 2), together with the monomer conversion over time. Figure 2 shows that when the reaction was carried out at 44 °C, both PdI and zeta potential do not stabilize when compared to the results obtained at 70 °C. This suggests that the macroRAFT agent does not seem to be leaving the growing polymer particles to reinitiate the polymerization. Hence, the use of a sacrificial macroRAFT agent might have been required to ensure the living behavior of the polymerization.

The average molar mass of these copolymers was characterized by GPC-SEC analysis (Table 1). Even though only one peak was detected for the sample prepared at 70 °C, the dispersity (Đ) was 1.66, which is too high for controlled reversible deactivation radical polymerizations. The GPC-SEC results also confirm the low monomer conversion obtained namely for the copolymerization carried out at 44 °C, but in this case, the Đ = 1.27 indicates a controlled polymerization. As regards the discrepancy between the degree of polymerization (DP) and the average molar masses obtained gravimetrically and by GPC-SEC, this was naturally expected due to the major differences between the two methods. However, in the present study, the discrepancy may have been exacerbated by the fact that the calibration curve for GPC-SEC analysis was performed using polystyrene standards. Moreover, it should be stressed that the value for DP in the case of the copolymers corresponds to that associated with the chain length reached upon chain extension (DP_MMA:BA_). The results show that carrying out chain extension using emulsion polymerization under monomer starved conditions and at a low temperature compromises the yield of polymerization.

### 4.3. Preparation of Copolymer@Au NPs at 44 °C

Two types of Au nanostructures have been prepared after establishing the polymerization conditions for using the P(PEGA_40_)-TTC macroRAFT agent. One of such Au nanostructures comprises the use of the macroRAFT agent P(PEGA_40_)-TTC to yield the copolymer@Au NPs without azide moiety. The second one uses a mixture of this macroRAFT agent with the macroRAFT agent modified with the azide group (2 macroRAFT:1 *N3*-macroRAFT) to yield *N3*-copolymer@Au NPs. The [2 macroRAFT:1 *N3*-macroRAFT] molar ratio was selected in order to add the azide functionality to the nanostructure without significant perturbation of the emulsion copolymerization. Notice should be made that even though the determination of the amount of azide moiety per NP was not possible during the adsorption step, the solution of *N3*-macroRAFT agent was added dropwise to the colloidal Au NPs and stirred 2 h prior to the addition of the solution of macroRAFT agent (without azide) to ensure that all the *N3*-macroRAFT was adsorbed. Specifically, the main objective was to keep some carboxylate anions available to provide colloidal stabilization by electrostatic interactions and also to avoid crowding the surface with azide moieties. The former was used as reference, and the characterization of this colloid is presented in the Supporting Information (Figures S3 and S4, Table S2), whilst the characterization of the azide functionalized nanostructures is discussed below.

**Figure 3:**
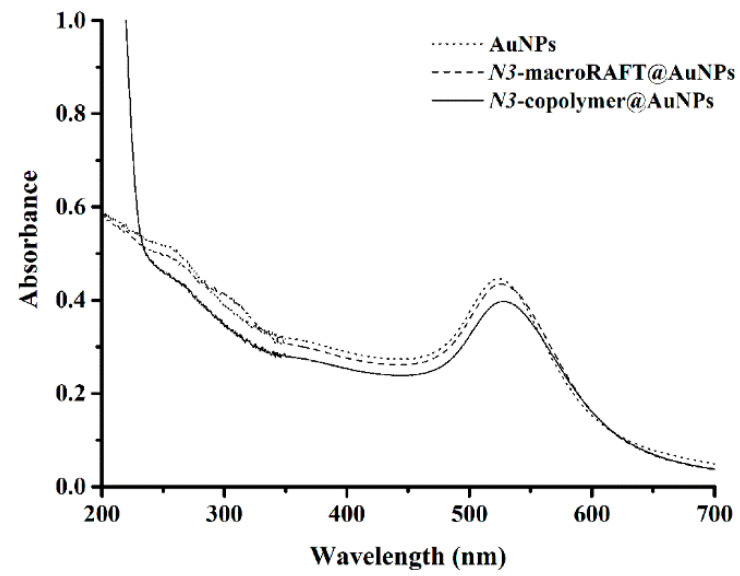
UV-Vis spectra of Au nanostructures before and after emulsion copolymerization at 44 °C, with azide moiety.

Figure 3 shows for the Au NPs, a first shift of λ_LSPR_ from 523 nm to 526 nm, and then to 529 nm, after adsorption of the macroRAFT agents onto Au NPs and after the chain extension from the *N3-*macroRAFT@Au NPs respectively (see λ_LSPR_ values in Table 2). Moreover, the increase of the absorbance band around 200–250 nm is associated with the presence of the hydrophobic block, indicating that chain extension from *N3*-macroRAFT@Au NPs had occurred, yielding *N3-*copolymer@Au NPs [15]. The DLS results (Table 2) show that after chain extension, there was a slight increase of the hydrodynamic average diameter (*z-average*), which is in line with our previous studies performed at 70 °C [16]. Furthermore, the small increase is in agreement with the low degree of polymerization registered for the corresponding copolymer.

**Figure 4:**
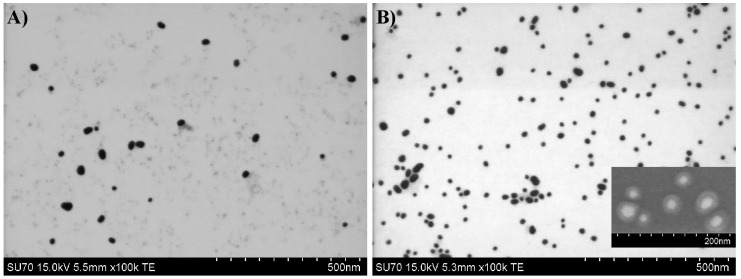
Scanning Electron Microscopy (SEM) in transmission mode (STEM) images of Au nanostructures prepared with the mixture 2macroRAFT:1 *N3*-macroRAFT agents (**A**) before and (**B**) after emulsion copolymerization. (**B**-*inset*) secondary electron imaging showing in detail the core–shell nanostructure.

As regards the surface charge of these nanostructures, Table 2 shows that after the adsorption of macroRAFT agents ([2 macroRAFT: 1 *N3*-macroRAFT] ratio) onto Au NPs, the zeta potential decreased from about −50 to −25 mV. This is associated with the replacement of the citrate ions at the Au NPs surface by the macroRAFT agents, which have one or two available carboxylic groups per chain, therefore decreasing the charge density of the NPs. Nevertheless, the colloid proved to be stable even after the copolymerization occurred because no aggregation was observed by monitoring the system using UV-Vis spectroscopy (Figure 3). Furthermore, electron microscopy images (Figure 4) showed that after chain extension, the polymer shell is well-defined (*inset* in Figure 4B and Figure S5 in the Supporting Information).

### 4.4. Biosensing Response

**Figure 5:**
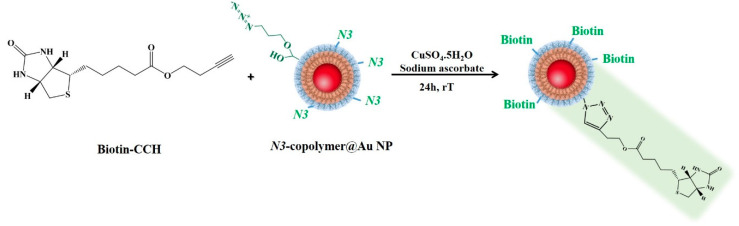
Schematic representation of biotinylation of *N3*–copolymer@Au NPs via the click chemistry reaction.

In order to evaluate the use of the above Au nanostructures for biosensing applications, the biotin–avidin (bioreceptor–biotarget) conjugate system was used here. For that purpose, the *N3–*copolymer@Au nanostructures were first biotinylated. Alkynated biotin (*biotin-CCH*, see the synthesis and characterization in the Supporting Information) was covalently bound to the *N3*–copolymer@Au nanostructures in the presence of CuSO_4_ and sodium ascorbate, forming a covalent 1,2,3-triazole linkage via click chemistry yielding *biotin*–copolymer@Au NPs (Figure 5).

After biotinylation, the UV-Vis spectrum showed a small shift in the λ_LSPR_ (Δλ ~ +1 nm) and no sign of aggregation (data not shown). The formation of the 1,2,3-triazole linkage, or consumption of azide, could not be detected due to the small amount of azide/triazole group in the Au colloid. However, as the click chemistry is known to be a very specific and highly efficient reaction [34], it was assumed that the reaction occurred yielding stable *biotin*–copolymer@Au NPs, and the optical biosensing response to the presence of the proteins of the ensuing Au nanostructures was studied. Moreover, notice should be made that the nanostructures were extensively rinsed to assess the efficiency of the click reaction. Additionally, this procedure was repeated in order to obtain enough material for the biosensing tests as well as for other ongoing studies, proving the reproducibility of this procedure.

The optical response of the biotinylated and non-biotinylated copolymer@Au NPs was assessed by adding to the colloidal nanostructures avidin, which is a protein that has high affinity toward biotin. BSA was used as a control because it is a protein that does not have affinity to biotin. Furthermore, since the addition of protein solution slightly dilutes the colloids, the same volume of phosphate buffer saline (PBS, pH 7.4, 10 mM) was added to all the colloids.

For comparison and as a control experiment, three Au colloids were tested: *biotin*–copolymer@Au NPs, the neat copolymer@Au NPs, and copolymer@Au NPs submitted to the conditions used in the click reaction (“click control”). The latter were copolymer@Au NPs (without the azide moiety), which were submitted to the same reaction conditions used to carry out the click reaction (i.e., alkynated biotin, CuSO_4_ and sodium ascorbate) and extensively rinsed, as described in the Experimental section to assess the efficiency of the click reaction. Solutions of avidin and of BSA (0.5 mg/mL) in PBS were added to the Au nanostructures, and after 10 min, the samples were characterized by UV-Vis spectroscopy. As a control of the dilution, PBS was also added to the three types of Au nanostructures.

**Figure 6:**
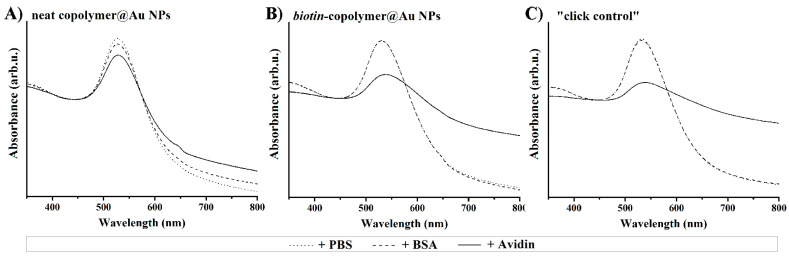
Visible spectra of the colloids in the presence of 10 μL of BSA and avidin, (**A**) *neat* copolymer@Au NPs, (**B**) biotin–copolymer@Au NPs, and (**C**) “click control”. PBS was used as dilution control, and used as blank. The spectra were normalized at 450 nm.

In Figure 6A the UV-Vis spectra are shown, and as it can be observed, a small decrease of the absorbance and a small shift in the λ_LSPR_ (Δλ = + 1 nm) occurred, especially in the presence of avidin, which indicates the occurrence of specific interactions. Nevertheless, nonspecific interactions seem to have occurred as well with the control of BSA, which might be assigned to a variety of interactions (electrostatic, H bonding, van der Waals, etc.) occurring between the nanostructures and the proteins [35,36]. Electrostatic interactions are very often referred as specific interactions present in such type of systems. In fact, at this pH (approximately 6, see Table 2), BSA presents a negative surface charge (pI = 4.7), while avidin is positively charged (pI = 10); hence, it will show a stronger response in these conditions. Figure 6B shows the visible spectra of the biotinylated nanostructures, which exhibit a strong decrease in the optical absorbance, the broadening, and the shift of the LSPR band (Δλ = +7 nm), revealing aggregation of the nanostructures resulting from the strong interaction between avidin and the biotinylated nanostructure. Surprisingly, the LSPR band of copolymer@Au NPs submitted to the “click reaction” (“click control” in Figure 6C) showed similar behavior in the presence of avidin. This result was not expected for the nanostructures without azide moieties, because the colloid had been extensively washed to remove unreacted species. This evidence indicates that alkynated biotin was not only covalently linked to the nanostructure by the specific click chemistry reaction with the azide but also adsorbed and/or was trapped in the polymer shell, contributing to the aggregation when in the presence of avidin. Therefore, intensive washing steps (precipitation/redispersion cycles) by centrifugation were performed in this colloid and, despite that, it still specifically aggregates in the presence of avidin (data not shown).

**Figure 7:**
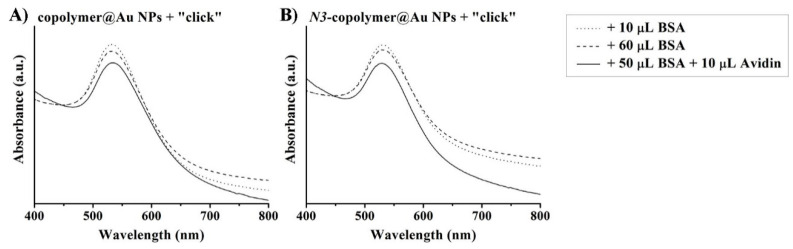
Visible spectra of (**A**) “click control” and (**B**) *biotin*–copolymer@Au NPs mixed with BSA (30 min) before adding avidin. The spectra were normalized at 450 nm.

In a second experiment, BSA was mixed with the nanostructures for 30 min before adding avidin to evaluate the possibility of minimizing nonspecific interactions, [37]. After 10 min in contact with avidin, the optical properties of the Au nanostructures were evaluated by visible spectroscopy (Figure 7). The use of a higher amount of BSA (60 µL) did not change the LSPR band, as compared with the 10 µL tested in the first experiment. Interestingly, the visible spectra of these nanostructures in contact with BSA and then with avidin showed that there was no broadening of the LSPR band, unlike the visible spectra in Figure 6, suggesting a different aggregation profile. In other words, BSA interacted with the Au nanostructures via nonspecific interactions without inducing aggregation and stayed at the surface of the nanostructures. Hence, when avidin was added, aggregation was induced due to the specific interaction with biotin. However, due to the presence of BSA (dimensions: 4 × 4 × 14 nm^3^ [38]) at the surface of the nanostructures, the gold cores in the aggregates were more apart from each other, so the λ_LSPR_ did not shift neither suffered a broadening, as was observed in the colloids without BSA; even so, some aggregation occurred.

### 4.5. Langmuir–Blodgett Studies at the Air/Water Interface

The adsorption and/or trapping of biotin by the polymer shell was not expected as discussed before. Hence, considering that Langmuir monolayers have been very successfully used to unravel interactions of biomolecules as well as nanoparticles with monolayers at air/water interface, including the kinetics of such interactions [39], this approach was also followed here. In particular, these studies were carried out to better understand the copolymer–biotin–avidin interactions, namely in what regards the interaction between biotin adsorption and the trapping of biotin by the polymer shell as well as the kinetics of botin/avidin interaction. For this particular study, biotin (without alkyne function) was mixed with the copolymer (without the azide moiety) before spreading it on the water surface. As the monolayer of the copolymer prepared at 44 °C afforded an unstable monolayer at the air/water interface due to its low molar mass (DP_MMA:BA_ = 56), the copolymer prepared at 70 °C (DP_MMA:BA_ = 168) was used.

**Figure 8:**
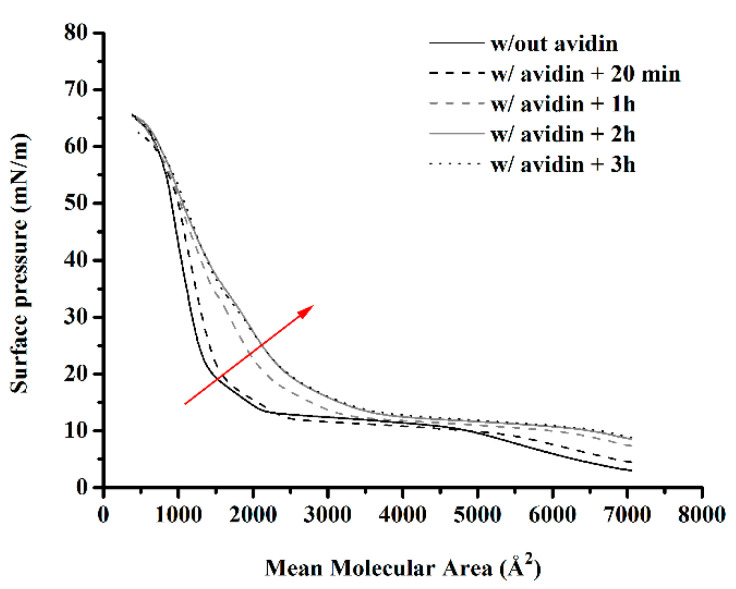
Langmuir surface pressure-area isotherm of P(PEGA_40_)-*b*-(MMA-*co*-BA)-TTC (VA-044 at 70 °C, 4 h) mixed with biotin, using aqueous avidin solution as the subphase and varying the contact time before compression starts.

The copolymer solution or copolymer solution containing biotin was spread on the water surface and later on the surface of an aqueous solution of avidin (0.5 mg/L). Upon solvent evaporation, the Langmuir monolayers were prepared by compressing the barriers. In a preliminary study, the Langmuir surface pressure-area isotherm (Figure S8 in Supporting Information) of the copolymer mixed with biotin showed a similar isotherm profile to that obtained for the neat copolymer. However, the liquid-condensed phase was formed at higher mean molecular areas, i.e., 1753 Å^2^, instead of 1670 Å^2^ for the neat copolymer. This result suggests that biotin was at the air/water interface and not just interacting with the hydrophilic PEGA chains, thus occupying space between the copolymer chains and shifting the liquid-condensed phase formation to a higher mean molecular area. No significant changes have been observed using an avidin solution instead of ultrapure water as the subphase. This could indicate that the biotin was not available to interact with avidin, i.e., avidin did not migrate to the air/water interface to interact with biotin, nor did biotin migrate to the subphase to interact with avidin. Assuming that time could be a parameter to be taken into consideration, the effect of contact time between the monolayer and the bioanalyte was evaluated. After spreading the Langmuir monolayers and waiting for 15 min to allow solvent evaporation, additional contact times of 20 min, 1 h, 2 h, and 3 h were tested prior to compression of the monolayer. The surface pressure versus area obtained for the different contact times considered are shown in Figure 8.

Although only a small difference can be observed after 20 min of contact between the monolayer and the subphase, upon 1 h, the isotherms showed a distinct profile, indicating that avidin was adsorbed onto the monolayer causing its expansion, especially when the monolayer was compressed to values smaller than 3000 Å^2^ where a transition from expanded to condensed liquid took place. These results indicate that this interaction is in fact time-dependent and is in agreement with what was observed in the colloids where biotin was not covalently bonded to the polymer shell.

As a final remark, it should be stressed that even though the biofunctionalization of copolymer@Au nanostructures via click chemistry is interesting for a variety of (bio)receptors in the specific case of biotin–avidin conjugate, the low solubility of biotin in water also revealed drawbacks. Nevertheless, it can be easily adapted to different bioreceptor–biotarget systems following a post-functionalization strategy of *N3*–copolymer@Au NPs. Alternatively, the use of biorecognition elements with higher solubility in water should be considered for the functionalization of *N3*–copolymer@Au nanostructures via click chemistry in order to ensure the complete removal of unbound material.

In the case of biotin or similar ligands (e.g., low solubility), the strategies to use click chemistry together with REEP methodology should be further studied in order to control the amount of bioreceptor anchored at the NPs surface. The results obtained using copolymer@Au NPs mixed with alkynated biotin (“click control”) have revealed potential to be applied to other (bio)molecules with low solubility in water. According to the work reported by Sasaki et al. [40], in connection with the preparation of poly[poly(ethylene glycol) methacrylates containing OH on the PEG side chains via ruthenium catalyzed living radical polymerization and subsequent functionalization with biotin for bioapplications, the use of OH-containing groups instead of their methylated counterparts seems a possible alternative. In any case, regardless of the synthetic route adopted, based on the results obtained in the present work, the covalent bond between biotin and the copolymer would need assessment.

These copolymer@Au nanostructures have also shown potential to be explored for drug carrier/delivery systems, since PEG is also known to have thermal responsiveness [41]. Moreover, the synthetic strategy outlined in this work also allows the incorporation of monomers containing fluorophores in well-defined positions along the polymer shell, which can be tuned to maximize photoluminescence quenching and thus afford more sensitive biosensors [42].

In what regards the limited monomer conversion values achieved due to the low reaction temperature and the use of monomer starved conditions required by the presence of azide moieties and REEP respectively, both can be overcome in the light of the results reported by Farinha et al. [19], the work of Stenzel [43], or alternatively the use of a redox initiation system as proposed by Bai et al. [44].

Finally, it should be emphasized that functionalizing tailor-made stable gold nanostructures in physiological medium toward biosensing still continues to be a challenge [45]. Indeed, although the desired core–shell nanostructures were obtained, further studies should be performed to get a better understanding about the chain extension from the macroRAFT@Au NPs, for example, in order to control the thickness of the polymer shell, which can be important to the response of the colloid to an external signal (e.g., temperature).

## 5. Conclusions

Functional polymer@Au nanostructures, with a core–shell type structure, were prepared combining RAFT-assisted encapsulating emulsion polymerization (REEP) and click chemistry. Despite the low monomer conversion (around 30%) due to the need to carry out chain extension at 44 °C, the colloidal stability of the ensuing Au nanostructure was good.

Furthermore, the possibility to post-functionalize robust functional core–shell plasmonic nanostructures with any desired bioreceptor proves to be a very interesting strategy especially if the bioreceptors have good water solubility. In fact, considering the COVID-19 pandemic, this type of nanostructures can find potential application in rapid and simple test kits in the point of care [46].

Depending on the functionalities associated with the polymer shell, a variety of biosensors is envisaged, such as pH, temperature, and photoluminescence quenching. The biosensing tests proved that *biotin*-copolymer@Au NPs specifically respond to the presence of avidin, inducing aggregation and subsequently changing the LSPR band. This interaction was proven to be time dependent.

## Figures and Tables

**Table 1:** Theoretical and experimental ${\overset{-}{M}}_{n}$ and degree of polymerization of macroRAFT and block copolymers P(PEGA_40_)-*b*-(MMA-*co*-BA)*_n_*-TTC prepared with VA-044.

	NMR				GPC-SEC				
	${\overset{-}{M}}_{n}$		DP		${\overset{-}{M}}_{n}$	${\overset{-}{M}}_{w}$		Đ	DP
**P(PEGA)_40_-TTC**	20,104		41		12,177	15,362		1.26	25
	**Theoretical *^a^***		**Gravimetry *^b^***			**GPC-SEC**			
	${\overset{-}{M}}_{n}$	**DP_MMA:BA_**	**%**	${\overset{-}{M}}_{n}$	**DP_MMA:BA_**	${\overset{-}{M}}_{n}$	${\overset{-}{M}}_{w}$	**Đ**	**DP_MMA:BA_**
**70 °C controlled addition**	35,912	181	35	23,823	63	15,119	25,136	1.66	29
**44 °C controlled addition**	35,079	173	24	21,577	41	12,246	15,532	1.27	1

Theoretical and gravimetric calculations used the ${\overset{-}{M}}_{n}$ value of macroRAFT determined experimentally by NMR. ***^a^*** Theoretical values of ${\overset{-}{M}}_{n}$ and degree of polymerization (DP_MMA:BA_) for 100% hydrophobic monomer conversion. ***^b^*** ${\overset{-}{M}}_{n}$ and DP_MMA:BA_ estimated based on monomer conversion of hydrophobic monomers (%) determined by gravimetric analyses. (i.e., overall DP = 41 + 63 for the reaction carried out at 70 °C).

**Table 2:** λ_LSPR_, DLS, and zeta potential (ζ) measurements of Au nanostructures before and after emulsion copolymerization at 44 °C, with azide function. Au NPs: colloidal gold nanoparticles.

	Au NPs	*N3*-MacroRAFT@Au NPs	*N3*-Copolymer@Au NPs
**λ_LSPR_ (nm)**	523	526	529
***z-average* (nm)**	16.3	26.6	27.9
**PdI**	0.573	0.561	0.531
**ζ (mV)**	−49.3 ± 1.8	−25.1 ± 1.9	−26.4 ± 1.2
**pH**	5.6	6.9	5.7

## Supplementary Materials

The following are available online at https://www.mdpi.com/2073-4360/12/7/1442/s1, Table S1: Experimental conditions used in the synthesis of the macroRAFT agents, Figure S1: ATR-FTIR spectra of P(AA_2_-*co*-PEGA_40_)-TTC before and after functionalization with the azide moiety, Figure S2: Monomer conversion (gravimetric analysis) and hydrodynamic diameter (*z-average*, DLS measurements) of aliquots withdrawn during the batch copolymerization of MMA:BA (10:1 *w*/*w*) in the presence of P(PEGA_40_)-TTC using the initiator VA-044 at (A) 70 °C and at (B) 44 °C, Figure S3: UV-Vis spectra of Au nanostructures before and after emulsion copolymerization at 44 °C, without azide functional groups, Table S2: λ_LSPR_, DLS and zeta potential (ζ) measurements of Au nanostructures before and after emulsion copolymerization at 44 °C, without azide functional groups, Figure S4: STEM images of Au nanostructures prepared only with P(PEGA_40_)-TTC, without azide moiety, Figure S5: SEM images of Au nanostructures prepared with the mixture 2macroRAFT:1 *N3*-macroRAFT agents after emulsion copolymerization, Figure S6: Preparation of alkynated biotin, Figure S7: ^1^H-NMR of alkynated biotin in DMSO-*d*_6_, Figure S8: Langmuir surface pressure-area isotherms of the copolymer P(PEGA40)-*b*-(MMA-*co*-BA)-TTC (VA-044 at 70 °C) mixed with biotin before spreading and using ultra-pure water as subphase or an avidin solution (0.5 mg/L in ultra-pure water).
