# Supplementary material for: Biofunctional Polymer Coated Au Nanoparticles Prepared via RAFT-Assisted Encapsulating Emulsion Polymerization and Click Chemistry
Source: Polymers (Basel). 2020 Jun 27;12(7):1442. doi: 10.3390/polym12071442 (PMC7408358; doi:10.3390/polym12071442)
Supplement: Supplementary file 1 [file polymers-12-01442-s001.pdf]

Supporting Information

Biofunctional polymer coated Au nanoparticles prepared via RAFT-assisted encapsulating emulsion polymerization and *click chemistry*

Sónia O. Pereira, Tito Trindade, Ana Barros-Timmons

Department of Chemistry
CICECO- Aveiro Institute of Materials
University of Aveiro
3810-193 Aveiro
Portugal

Synthesis of macroRAFT agents: P($PEGA_{40}$)-TTC and P(AA_2-*co*-$PEGA_{40}$)-TTC

Table S1. Experimental conditions used in the synthesis of the macroRAFT agents.

macroRAFT agent	P($PEGA_{40}$)-TTC	P(AA_2-*co*-$PEGA_{40}$)-TTC
**Monomer**	PEGA	AA + PEGA
**[TTC-A] (mM)**	86	50
**[monomer]/[TTC-A]**	44	2 (AA) + 41 (PEGA)
**[TTC-A]/[ACPA]**	10	10
**Solvent**	ethanol	ethanol
**Time (hours)**	4	3 (AA) + 4 (PEGA)
**Temperature (ºC)**	70	70
$\bar{M}_{n\ (theoretical)}$ **(g/mol)**	21387	19990
%Conversion **(^{1}H-NMR)**	94	17 (AA) + 91 (PEGA)*
$\bar{M}_{n\ (exp\_NMR)}$ **(g/mol)**	20104	18291
**DP** $_{(exp\_NMR)}$	41	1 (AA) + 36 (PEGA)
$\bar{M}_{n\ (exp\_GPC)}$ **(g/mol)**	12177	11318
**Đ**	1.26	1.25

- This value corresponds to the conversion of PEGA monomer. The conversion of AA estimated by ^{1}H NMR after 3 h was 17%. However, this value must be taken with caution! Due to the very small quantities used in the polymerization of AA, the error associated in the determination of its conversion may be quite high!

Characterization *N3*-macroRAFT agent

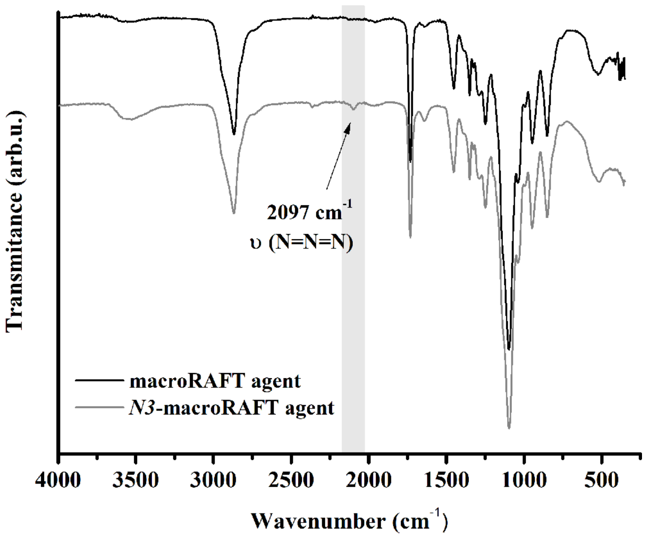

Figure S1. ATR-FTIR spectra of P(AA_2-*co*-$PEGA_{40}$)-TTC before and after functionalization with the azide moiety.

Determination of the number of azide moities per macroRAFT agent

The determination of the number of azide moieties per macroRAFT agent chain was estimated by the integration of the chemical shift of the protons from the 3-azido-1-propanol, using as reference the protons of $–CH_3$ from *Z*-group of the macroRAFT agent (see the NMR spectra in **Figure 1**). The next equation was used:

$$n_{H^c} = \frac{n_{H^{ref}} \times I_{H^c}}{I_{H^{ref}}}$$

n_{H^c}- number of protons that corresponds to the chemical shift of ***c***
$n_{H^{ref}}$- number of protons that corresponds to the chemical shift of the ***reference***
I_{H^c}- integration of chemical shift of ***c***
$I_{H^{ref}}$- integration of chemical shift of the ***reference***

The software MestReNova© was used to analyze the ^{1}H-NMR spectra. The chemical shift of the protons of reference (marked in the spectrum with *, δ=0.84 ppm, in **Figure S1**) were integrated to 1 ($I_{H^{ref}} = 1$). This signal corresponds to 3 protons ($n_{H^{ref}} = 3$). The integration of the protons of ***c*** at the chemical shift 4.57 ppm was equal to 1.09 (I_{H^c}=1.09). Subsequently $n_{H^c} = 3.27$. But this chemical shift corresponds to a $–CH_2$-, which means that there is 1 or 2 azide moieties per chain. (If $n_{H^c} = 2$ would correspond to one azide per chain, if $n_{H^c} = 4$ would correspond to two azides per chain).

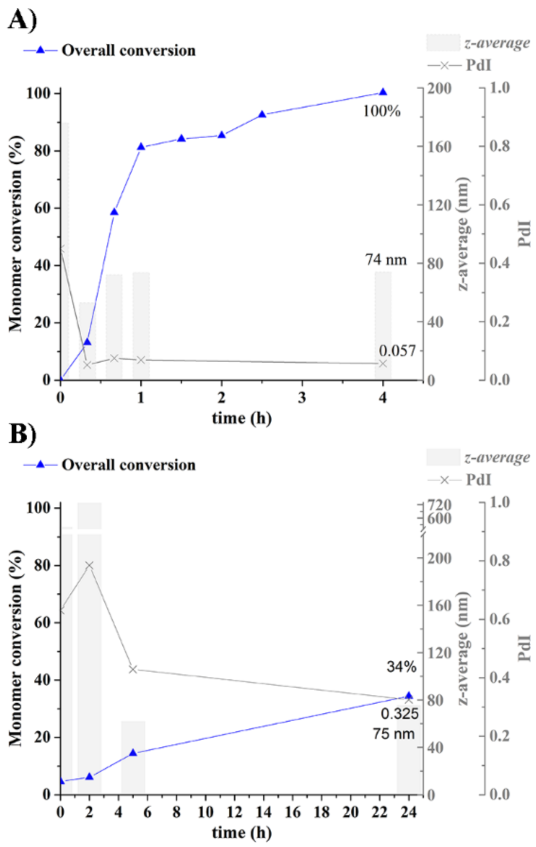

Figure S2. Monomer conversion (gravimetric analysis) and hydrodynamic diameter (*z*-average, DLS measurements) of aliquots withdrawn during the batch copolymerization of MMA:BA (10:1 w/w) in the presence of P(PEGA$_{40}$)-TTC using the initiator VA-044 at (A) 70ºC and at (B) 44ºC.

Characterization of copolymer@Au NPs prepared at 44ºC

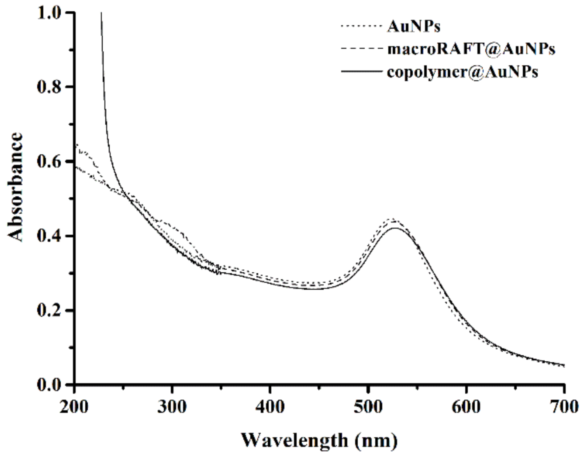

Figure S3. UV-Vis spectra of Au nanostructures before and after emulsion copolymerization at 44ºC, without azide functional groups.

Table S2. λ_{LSPR}, DLS and zeta potential (ζ) measurements of Au nanostructures before and after emulsion copolymerization at 44ºC, without azide functional groups.

	**Au NPs**	**macroRAFT@Au NPs**	**copolymer@Au NPs**
**λ_{LSPR} (nm)**	523	525	528
z-average (nm)	16.3	25.8	27.3
**PdI**	0.573	0.586	0.546
**ζ (mV)**	-49.3 ± 1.8	-25.7 ± 3.1	-31.8 ± 1.9
**pH**	5.6	6.9	5.6

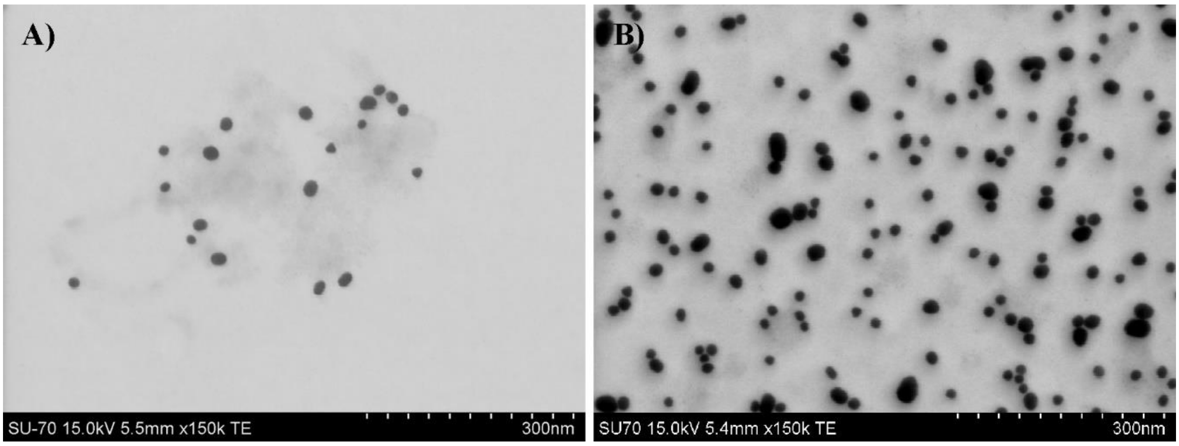

Figure S4. STEM images of Au nanostructures prepared only with P(PEGA$_{40}$)-TTC, without azide moiety, (A) before and (B) after emulsion copolymerization. In (B) the slight grey background around the Au NPs indicates the presence of the polymer, yielding the core-shell type structure copolymer@Au NPs

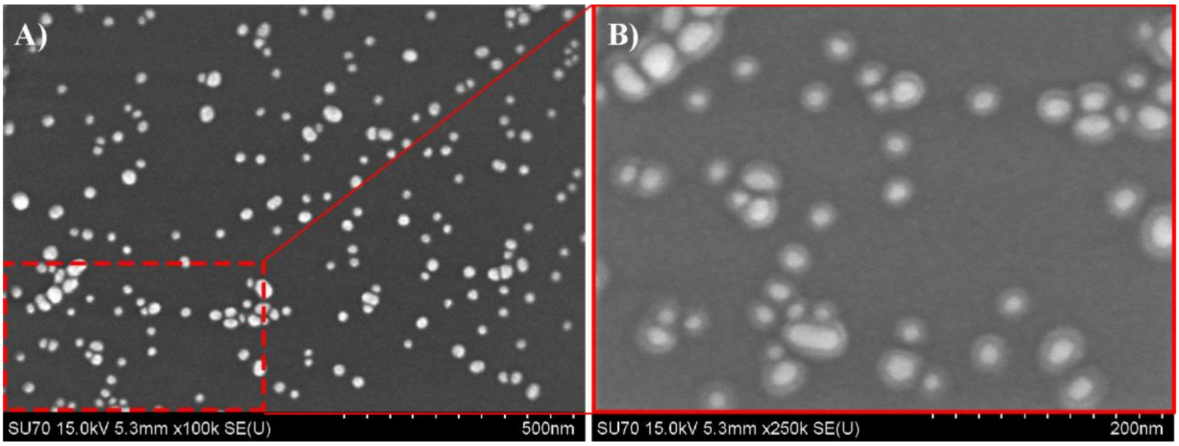

Figure S5. SEM images of Au nanostructures prepared with the mixture 2macroRAFT:1 *N*3-macroRAFT agents after emulsion copolymerization. Secondary electron imaging (A) of Figure 4B (in the main manuscript) and (B) higher magnification of the delimited red zone corresponding to the inset in Figure 4B.

Synthesis and characterization of alkynated biotin

Alkynated biotin (*biotin-CCH*) was prepared following the procedure described in [1]. An esterification reaction was promoted in DMF, using EDC and DMAP, between the carboxylic acid group from biotin and the hydroxyl group from 3-butyn-1-ol (**Figure S6**). The alkylated biotin was characterized by ^{1}H-NMR, see **Figure S7**.

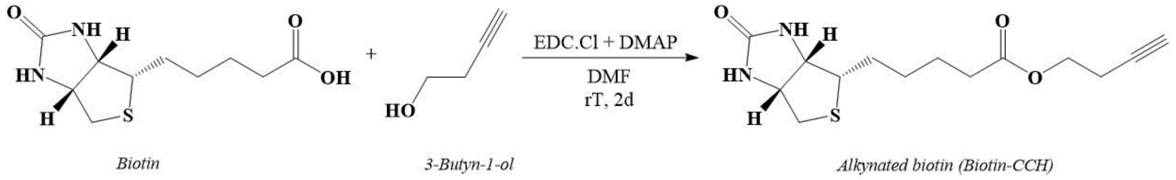

Figure S6. Preparation of alkynated biotin.

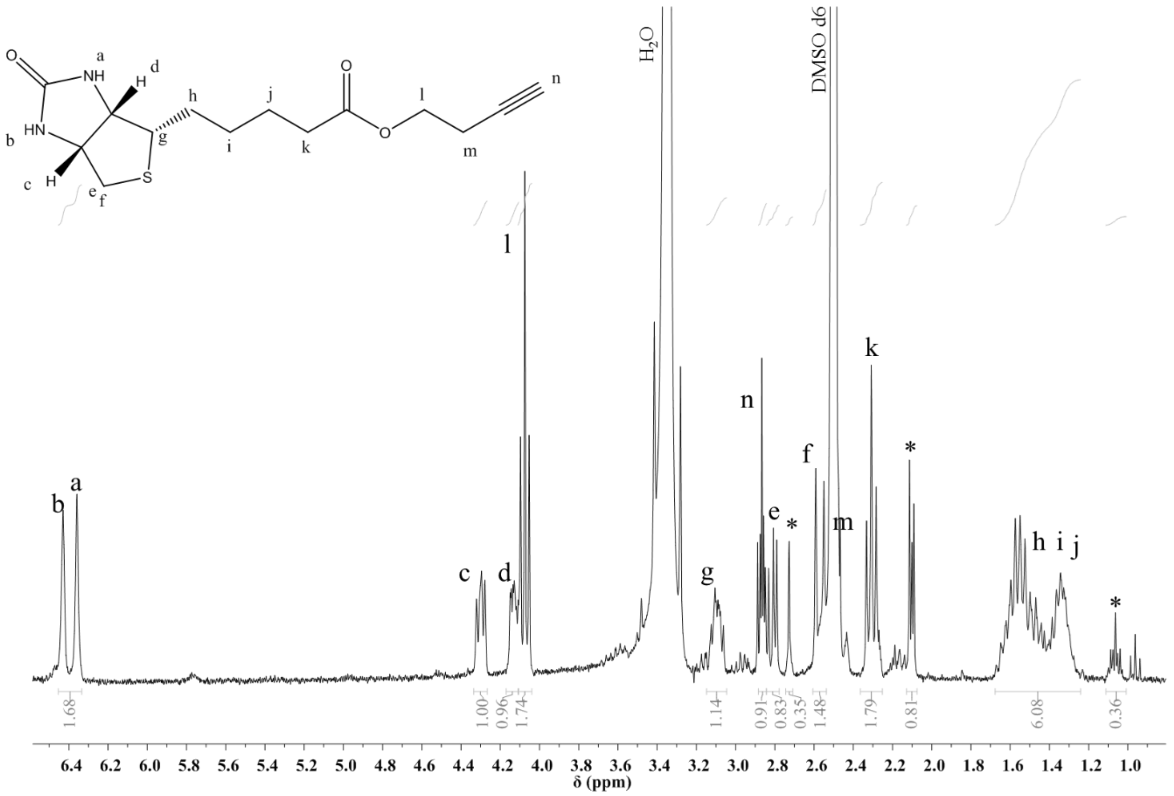

Figure S7. ^{1}H-NMR of alkynated biotin in DMSO-d_6. (* EDC residues).

Preliminary Langmuir-Blodgett studies at the air/water interface

These preliminary studies were performed using a NIMA 611 Langmuir-Blodgett trough. 50 µL of copolymer solution or copolymer containing biotin, as described in the main manuscript, were spread at the air/water interface using ultra-pure water or an aqueous solution of avidin (0.5 mg/mL) as subphase. After 15 min, to reach equilibrium (solvent evaporation), the Langmuir monolayers were prepared compressing the barriers at 15 cm^2/min. The corresponding isotherms are shown in **Figure S8**.

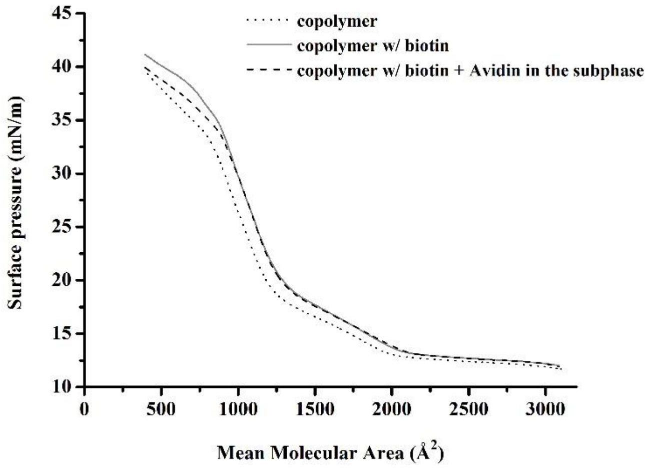

Figure S8. Langmuir surface pressure-area isotherms of the copolymer P($PEGA_{40}$)-*b*-(MMA-*co*-BA)-TTC (VA-044 at 70ºC) mixed with biotin before spreading and using ultra-pure water as subphase or an avidin solution (0.5 mg/L in ultra-pure water).

References

[1] D.J. Siegwart, J.K. Oh, H. Gao, S. a. Bencherif, F. Perineau, A.K. Bohaty, J.O. Hollinger, K. Matyjaszewski, Biotin-, Pyrene-, and GRGDS-Functionalized Polymers and Nanogels via ATRP and End Group Modification, Macromol. Chem. Phys. 209 (2008) 2179–2193. doi:10.1002/macp.200800337.
